# First external validity study of the Fagotti score in ovarian cancer

**DOI:** 10.1038/s41598-024-62568-0

**Published:** 2024-05-27

**Authors:** Sarah Aida, Mathieu Levaillant, Henri Azaïs, Marcos Ballester, Geoffroy Canlorbe, Pauline Chauvet, Tristan Gauthier, Cyrille Huchon, Yohan Kerbage, Martin Koskas, Lise Lecointre, Lobna Ouldamer, Émilie Raimond, Vincent Lavoué, Guillaume Legendre

**Affiliations:** 1 Department of Gynecology, University Hospital, 49100 Angers, France; 2 Department of Public Health, University Hospital, 49100 Angers, France; 3 Department of Gynecology and Breast Oncological Surgery, Georges-Pompidou European Hospital, APHP Centre, University of Paris, 75015 Paris, France; 4 Department of Gynecology, University Hospital, Diaconnesses La Croix Simon, 75012 Paris, France; 5 Department of Gynecology, University Hospital, APHP- La Pitié Salpêtrière, 75013 Paris, France; 6 Department of Gynecology, University Hospital, 63000 Clermont-Ferrand, France; 7 Department of Gynecology, University Hospital, 87042 Limoges, France; 8 Department of Gynecology, Lariboisière Hospital, AP-HP, University of Paris, 75010 Paris, France; 9 Department of Gynecology, University Hospital, 59000 Lille, France; 10 Department of Gynecology, Hospital, Bichat, Paris Cité University, 75018 Paris, France; 11 Department of Gynecology, University Hospital, 67200 Strasbourg, France; 12 Department of Gynecology, University Hospital, Bretonneau, 37044 Tours, France; 13 Department of Gynecology, University Hospital, 51100 Reims, France; 14 Department of Gynecology, University Hospital, 35033 Rennes, France

**Keywords:** Ovarian cancer, Metastasis

## Abstract

Epithelial ovarian cancer is mostly discovered at the stage of peritoneal carcinosis. Complete cytoreductive surgery improves overall survival. The Fagotti score is a predictive score of resectability based on peritoneal laparoscopic exploratory. Our aim was to study the inter-observer concordance in an external validation of the Fagotti score. An observational, prospective, multicenter study was conducted using the Francogyn research network. The primary outcome was inter-observer concordance of the Fagotti score. 15 patients in which an ovarian mass was discovered were included. For each patient, the first exploratory laparoscopy before any treatment/chemotherapy was recorded. This bank of 15 videos was subject to blind review accompanied by a Fagotti score rating by 11 gynecological surgeons specializing in oncology. A total of 165 blind reviews were performed. Inter-observer concordance was very good for the Fagotti score with an intraclass correlation coefficient (ICC) of 0.83 [95% CI 0.71; 0.93]. Inter-observer concordance for the adjusted Fagotti score, which accounts for unexplorable areas with extensive carcinomatosis, resulted in an ICC of 0.64 [95% CI 0.46; 0.82]. According to the reviewers, the three least explorable parameters were mesentery involvement, stomach infiltration and liver damage. The ICC of the explorable Fagotti score, i.e. score with deletion of the parameters most often unexplored by laparoscopy, was 0.86 [0.75–0.94]. This study confirms the reproducibility of the Fagotti score during first assessment laparoscopies in cases of advanced ovarian cancer. The explorable Fagotti score has an equivalent or better inter-observer concordance than the Fagotti score.

## Introduction

Epithelial ovarian cancer (EOC) is the seventh most common cancer in women in order of frequency and the fifth leading cause of cancer death in developed countries^1^. It is the deadliest female cancer. 75% of patients are diagnosed with an advanced stage (stages III-IV of the 2014 FIGO classification)^2^, with peritoneal metastases^3^.

The overall 5-year survival rate is about 45%^2,3^. 313,959 new cases were diagnosed worldwide in 2020 (incidence rate estimated at 6.6/100,000) with 207,252 deaths (mortality rate estimated at 4.2/100 000)^1^. On average, one in seventy women will develop EOC^4^. Its treatment is based on surgery and chemotherapy, mainly by means of taxanes and platinum salts. In addition to conventional treatment, some new treatments such as anti-PARP treatment lead to a new prognosis in patients with DNA double-strand repair deficiency^5^.

Complete macroscopic cytoreductive surgery (CRS) is the most important prognostic factor with respect to overall survival^6–8^. The goal is to achieve no residual disease following CRS, meaning without any macroscopically visible lesion. If complete CRS is not feasible, neoadjuvant chemotherapy is recommended with the aim being to perform interval CRS after 3–4 chemotherapy cycles^9^. The assessment of surgical resectability is paramount. Surgery resulting in residual tumor disease does not offer any therapeutic effect or greater likelihood of survival^8^.

To predict the probability of achieving complete CRS, different approaches were studied: biological markers with CA-125, computed tomography-based imaging with Bristow classification^10–12^ and scores evaluated during surgery. Sugarbaker's Peritoneal Cancer Index is a score used in median laparotomy^13^. In 2006, a laparoscopic score with an optimal resectability threshold was proposed by the team of Fagotti et al.^14^.

This score was established in reference to optimal debulking surgery (residual tumor < 1 cm) but was also validated for complete cytoreduction surgery (CC0) by the team of Petrillo et al.^15^.

The use of a scoring system is recommended as a common practice for assessing patients eligible for initial surgery in the advanced stages of epithelial ovarian cancer (EOC). In fact, it may play a role in guiding the evaluation and ultimately triage patients for primary management. However, there is no universally accepted scoring system endorsed by the ESMO-ESGO consensus conference (Level III, Grade C)^16^. Presently, the Fagotti score stands out as one of the most frequently utilized scoring systems in current practice, backed by several European professional societies (CNGOF, ESMO-ESGO)^9,17^

Despite its widespread use, only the reproducibility of Fagotti's score through proofreading by the promoting team that developed it has been validated so far^18^. It should be noted that this score is sometimes incomplete, with areas that cannot be explored by surgeons. No inter-observer concordance exists in the literature to verify the identical assessment of the same patient by different teams. Our main objective was to evaluate the inter-observer concordance of the Fagotti score in an external validation.

## Materials and methods

### Study methodology

A prospective multicenter non-interventional study was conducted from January 2021 to June 2021 within the FRANCOGYN network.

### Ethical committee exemption

The protocol experimental was approved by a licensing committee “Ethics and Research Committee in Obstetrics and Gynaecology France”. The authorization number was CEROG 2020 GYN-1106. All methods were performed in accordance with the relevant guidelines and regulations. This study was in compliance with the Data Protection Act MR-004 under CNIL registration number ar20-01209v0. All patients gave their informed consent. A secure computer storage server was used.

**Figure 1:**
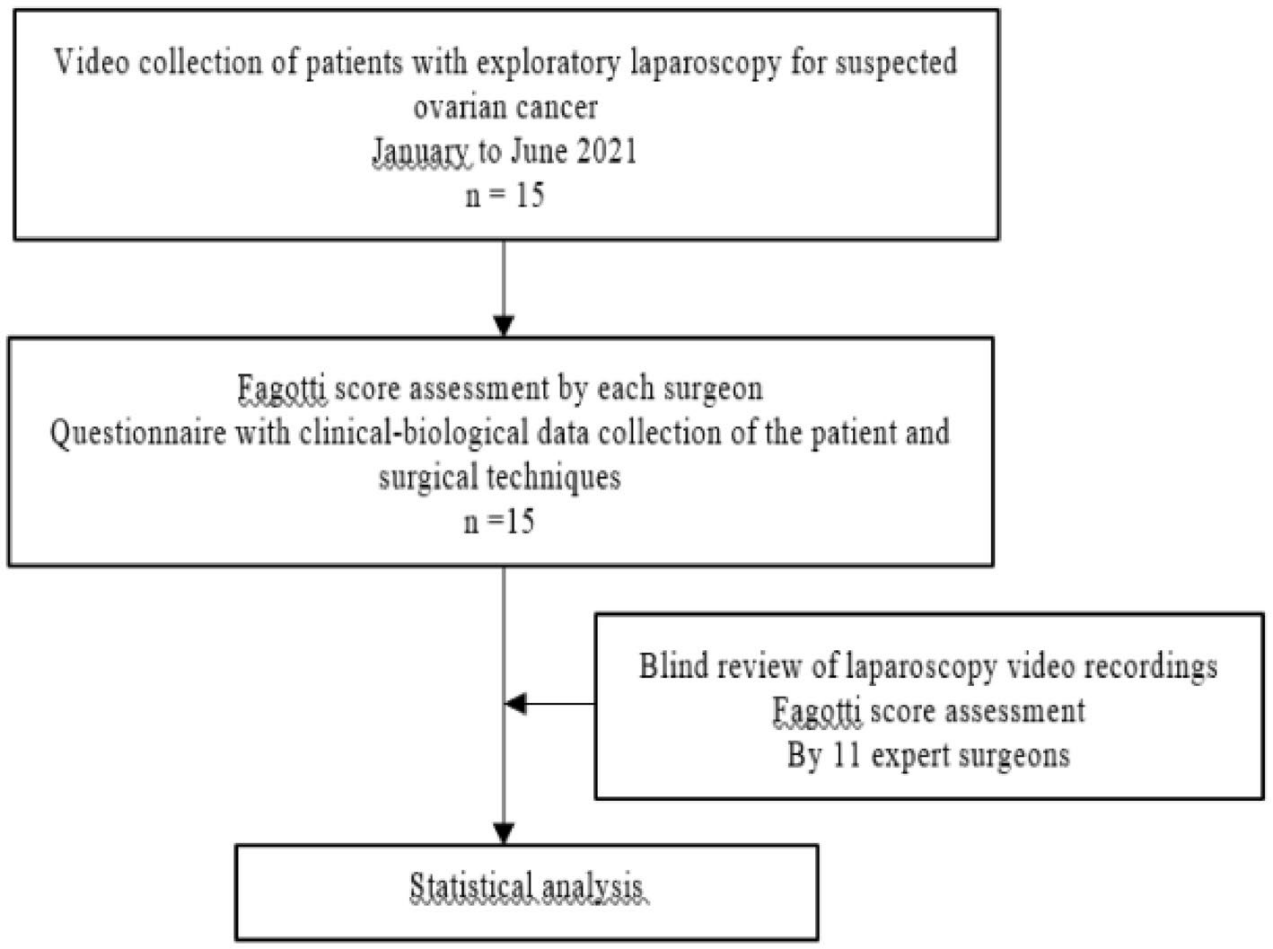
Flow-Chart.

Each center recorded the standardized laparoscopic exploration of one or two peritoneal carcinomatosis in a patient with EOC in initial assessment surgery. The inclusion criteria were as follows: all adult and consenting women having no contraindication to abdominopelvic surgery and having an exploratory laparoscopy prior to the discovery of a carcinomatosis with pathological confirmation of EOC. 15 videos from 11 different centers were collected (Appendix 1, supplementary material). Surgeons had to complete a questionnaire that included the operator's Fagotti score, information on surgical and clinical-biological technique, and imaging tests. From July to August 2021, surgeons had to review the video bank and assign a Fagotti score to each video. This was carried out blind to the clinical characteristics of each patient. 11 readings were collected. The design of the study is shown in Fig. 1.

The were standardized with quadrant-by-quadrant abdominal exploration. The surgeons were asked to film clockwise quadrant by quadrant: left iliac fossa, pelvis, right iliac fossa, right flank, right diaphragmatic cupola with liver visualization, epigastric region, left diaphragmatic cupola, central region. During exploration of each quadrant, the various parameters of the Fagotti score are visualized as far as possible. The quality of each video was rated on a numerical satisfaction scale from 0 to 10 by the reviewers. This evaluation was subjective, based on image sharpness, visibility, brightness and the overall appearance of the video.

### Definitions

- Fagotti score: Score defined previously by Fagotti et al.^14^, based on the laparoscopic evaluation of seven anatomical areas (peritoneum, diaphragmatic cupolas, mesentery, omentum, digestive tract, stomach, liver). It describes the intra-abdominal diffusion of the disease. The authors estimated the surgical outcome by means of calculating a global predictive value index. Each item is assigned a predictive index value of 2, with a total score of 8 or higher indicating near-zero resectability. Specifically evaluations included the following: (1) peritoneal carcinomatosis, a score of 2 was allotted only to the patients with massive peritoneal involvement as well as with a miliary pattern of distribution; on the contrary, the score was 0 in the case of carcinomatosis involving limited area (as along the paracolic gutter or the pelvic peritoneum) being surgically removable by peritonectomy; (2) diaphragmatic disease: a score of 2 was agreed in the case of widespread infiltrating carcinomatosis or confluent nodules to the utmost part of the diaphragmatic surface; (3) mesenteric disease: a score of 2 was granted when large infiltrating nodules or an involvement of the root of the mesentery were supposed on the basis of limited movements of the various intestinal segments. On the other hand, small nodules potentially treated by argon beam coagulator (ABC) were not considered for scoring; (4) omental disease: a score of 2 was allotted when tumor diffusion was observed along the omentum up to the large stomach curvature, whereas isolated localization were excluded; (5) bowel infiltration: a score of 2 was agreed in the case that a bowel resection was assumed or when extended carcinomatosis on the ansae was observed; (6) stomach infiltration: a score of 2 was granted when an obvious neoplastic involvement of the gastric wall was observed; and (7) liver metastases: a score of 2 was allotted in the case of surface lesions larger than 2 cm.
- Modified Fagotti score: Score out of 8 as defined by Tenon's team^19^. This modified score was created by selecting 4 of the 7 parameters: diaphragmatic carcinosis, mesenteric retraction, stomach infiltration, liver metastases.
- A modified score of ≥ 4 was associated with suboptimal cytoreduction with a sensitivity, specificity, PPV, NPV, and accuracy of 35, 100, 100, 43, and 56% respectively compared to 46, 89, 89, 44, and 60% with the laparoscopy-based score of ≥ 8^20^.
- Adjusted Fagotti score: Score where each non-explorable parameter is regarded as being positive for carcinosis, with + 2 added to the rating.
- Explorable Fagotti score: The Fagotti score parameters that are most frequently unexplorable by laparoscopy have been removed.

### Primary and secondary outcome

The primary outcome of the study is inter-observer concordance in the Fagotti score. In the event of discrepancy, the variability was analyzed on the basis of the characteristics of the patients, the FIGO stage and the surgical technique used. The management of missing items from the score was taken into account in our secondary outcome. Non-explorable parameters were processed using different approaches to weighting: the adjusted Fagotti score and the explorable Fagotti score. The modified Fagotti score proposed by the Tenon team was also calculated. The secondary outcome was to establish which score had the highest inter-observer concordance by taking into account missing data.

### Statistical analysis

Fagotti score concordance was assessed by means of a bidirectional mixed intraclass coefficient (ICC) with two-way random effects. The confidence interval was 95%. R software, RStudio Team (2023) RStudio: Integrated Development for R. RStudio, PBC, Boston, MA, was used for statistical analysis. Four categories are established for interpreting the CCI^21^: below 0.50: low; between 0.50 and 0.75: average; between 0.75 and 0.90: good; above 0.90: excellent. We collected information about the patient's age, BMI, history of abdominal surgery, whether or not an imaging test was performed before the procedure, and its results. We used Excel software, Microsoft Excel Mac version 16.8 (2023), for data collection and Word, Microsoft Word pour mac version 16.8 (2023), for tables.

## Results

At all data of 15 patients were recorded. The characteristics of the 15 patients included in the study are described in Table 1.

**Table 1:** Patient characteristics.

Variables	Data
Age (years), *median y [interquartile range]*	57 [57.5–75]
BMI, *median y [interquartile range]*	20.7 [20.6–24.6]
< 25	11 (73.3%)
25–30	1 (6.7%)
≥ 30	3 (20%)
History of abdominopelvic surgery	9 (60%)
Type of scar surgical history*	
Laparotomy-median	1 (11.1%)
Pfannenstiel	3 (33.3%)
Laparoscopy	6 (66.6%)
FIGO stage	
I-II	1 (6.7%)
III	12 (80%)
IV	2 (13.3%)
CA 125 preoperative (UI/ml), *median y [interquartile range]*	500 [218–1068]
≤ 500	9 (60%)
> 500	6 (40%)
Preoperative imaging	
Abdominopelvic CT	14 (93.4%)
Pelvic MRI	9 (60%)
PET-CT 18 FDG	3 (20%)

*1 patient had an history of laparoscopy and laparotomy.

The median age was 57 years [57.5–75]. 80% of patients were stage III according to the FIGO classification. The median CA 125 was 500 IU/ml [218–1068]. Data on the surgical laparoscopy technique are summarized in Table 2.

**Table 2:** Surgical characteristics.

Variables	Data
Channel used	
Open laparoscopy	12 (80%)
Direct insufflation trocar	1 (6.7%)
Palmer's needle	2 (13.3%)
Type of optics used	
5 mm	1 (6.7%)
10 mm	14 (93.3%)
Position of the trocars	
Midline	14 (93.3%)
Right Iliac Fossa	4 (26.7%)
Left Iliac Fossa	1 (6.7%)
Right flank	1 (6.7%)
Number of trocars used in addition to that of the optics	
1	10 (66.7%)
2	5 (33.3%)
Realization of a cytology of peritoneal fluid	14 (93.3%)
Location of biopsies performed	
Right flank	7 (46.7%)
Left flank	6 (40%)
Retro-uterine	2 (13.3%)
Vesico-uterine peritoneum*	3 (20%)
Epiploic cake	6 (40%)
Fallopian tube	4 (26.7%)
Ovaries	3 (20%)
Uterine serosa	2 (13.3%)
Parieto-colic gutter	6 (40%)
Anterior parietal pelvic peritoneum	2 (13.3%)
Diaphragmatic dome	2 (13.3%)
Other: mesenteric nodule	1 (6.7%)

*including a pre-bladder because of hysterectomy antecedent.

In 80% of cases the first preferred route was open laparoscopy, with 10 mm optics. Only one trocar was generally used in addition to optics (66.7%). It is interesting to note that 4 trocars were positioned in the right iliac pit, and 1 in the left iliac pit. These trocars were placed to pull and facilitate visualization of difficult areas. Biopsies were mainly performed on the flanks, omentum and parieto-colic gutter (40%).

We also collected characteristics of the surgeon reviewers (Table.S1). The median number of surgical procedures performed on EOC each year was 10^5–20^. The median number of years of experience among the surgeons was 8^6–11^. If a Fagotti criterion was considered non-assessable, 27.2% of surgeons considered it to be met, 9.1% as unmet. 63.6% responded that they considered this criterion to be met or unmet depending on the context (imaging, tumor load).

The quality of the laparoscopy videos was considered satisfactory to allow the Fagotti score to be rated at 86.8% [19.2] on average. The surgeons judged 11 videos out of 15 to be of very good quality, 8 of which were 100% satisfactory. 2 videos were rated as being of average quality with 63% and 80% satisfaction., and 2 videos were judged to be of poor quality with 45% satisfaction.

Data from the Fagotti score assessment by surgeon reviewers are shown in Table 3.

**Table 3:** Score assessment by surgeon reviewers.

Fagotti score parameters	Number of responses	Absent (0 point)		Present (2 points)		Not explorable		Fleiss' κappa coefficient	Κappa* p* value
		Number of responses	(%)	Number of responses	(%)	Number of responses	(%)		
Peritoneal carcinosis	165	27	16.36	138	83.64	0	0.00	0.682	*p *< 0.001
Carcinoma of the diaphragmatic cupola	165	21	12.73	144	87.27	0	0.00	0.234	*p* < 0.001
Carcinoma of the mesentery	165	57	34.55	76	46.06	32	19.39	0.462	*p* < 0.001
Epiploic carcinoma	165	26	15.76	130	78.79	9	5.45	0.392	*p* < 0.001
Affecting the digestive tract	165	28	16.97	122	73.94	15	9.09	0.502	*p* < 0.001
Stomach infiltration	165	95	57.58	35	21.21	35	21.21	0.431	*p* < 0.001
Liver metastases	165	97	58.79	30	18.18	38	23.03	0.122	*p* < 0.001

A total of 165 responses were received (15 blind laparoscopy assessments per 11 experts). The two parameters of the Fagotti score most often deemed to be “present” were carcinomatosis of the diaphragmatic cupola (87.27%) and peritoneal carcinosis (83.64%). Conversely, liver metastases (58.79%) and stomach infiltration (57.58%) were mainly assessed as "absent". Some parameters of the Fagotti score were listed as "not explorable". These parameters were mainly the liver with the presence or absence of liver metastases (23.03%), stomach infiltration (21.21%) and mesentery involvement (19.39%). The item-by-item coincidence rate was calculated using Fleiss' Kappa on a categorical approach based on the reviewers' classification of each lesion as present, absent or not explorable. In terms of interpretation, this is similar to Cohen's classic kappa: values > 0.75 correspond to excellent agreement, values < 0.40 to poor agreement, and values between [0.40–0.75] to fair to good agreement. Three items obtained poor agreement: diaphragmatic cupola, liver lesions, omentum carcinosis.

**Figure 2:**
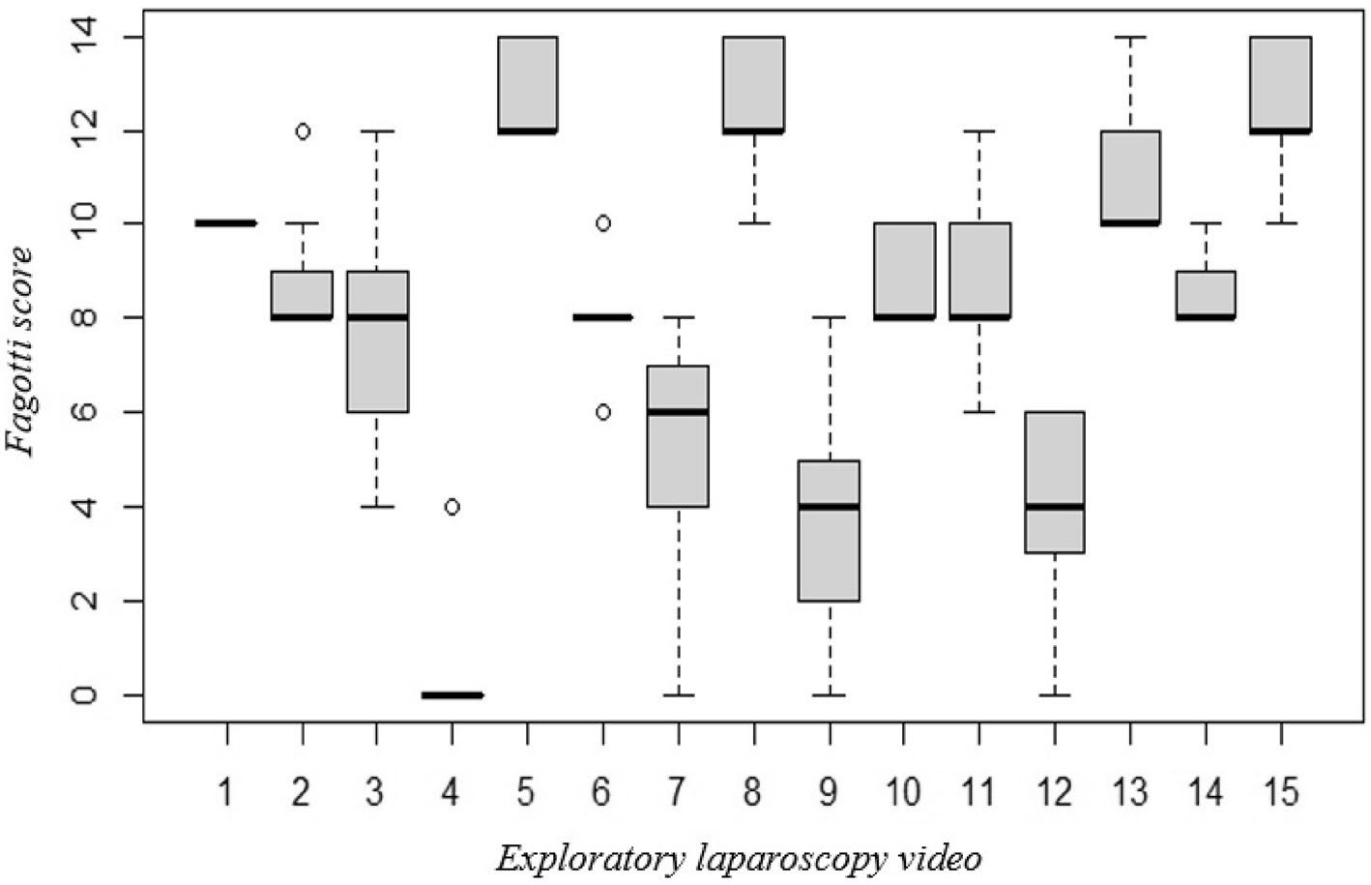
Box plot—analysis of the inter-observer correlation in Fagotti score between different assessing surgeons.

The correlation in Fagotti score scoring between different assessing surgeons is illustrated in Fig. 2.

For example, for video 1, there was a median score of 10, with all the assessors assigning the same score. There were also disparities in the scoring, with video 3 serving as an illustration. In this case, the median score was 8 with an interquartile between 6 and 9 and adjacent minimum and maximum values at 4 and 12. In general, it is observed that for the high values of the score (≥ 10) the interquartile gap was reduced and there were no outliers.

The intra-class correlation coefficient is shown in Table 4. Inter-observer concordance for Fagotti's score was good, with an ICC of 0.83 [0.71; 0.92]. The CCI of the adjusted Fagotti score was 0.64 with an extended 95% confidence interval [95% CI, 0.46; 0.82] or a weak to moderate concordance. The modified Fagotti score also displayed average concordance, with an ICC of 0.72 [0.56–0.87]. The explorable Fagotti score had good to excellent inter-observer concordance with the highest ICC at 0.86 for a narrow confidence interval [0.75–0.94]. We have redone the statistical analyses by excluding the early-stage patient and the results did not significatively differs. The Fagotti score remained with a rather good agreement ICC of 0.76 [95% CI, 0.61; 0.90] and the explorable Fagotti score ICC of 0.74 [95% CI, 0.58; 0.89].

**Table 4:** (a) The intra-class correlation coefficient. (b) The intra-class correlation coefficient excluding early-stage video.

	n	ICC	95% CI
(a)			
Fagotti score	11	0.83	0.71–0.92
Adjusted Fagotti score	11	0.64	0.46–0.82
Modified Fagotti score	11	0.72	0.56–0.87
Explorable Fagotti score	11	0.86	0.75–0.94
(b)			
Fagotti score	10	0.76	0.61–0.90
Adjusted Fagotti score	10	0.56	0.37–0.78
Modified Fagotti score	10	0.66	0.48–0.84
Explorable Fagotti score	10	0.741	0.58–0.89

## Discussion

We report here, to our knowledge, the first study assessing the inter-observer concordance of the Fagotti score, outside of the publications of the Fagotti team itself. Inter-observer concordance within our Fagotti score study was good, with an ICC of 0.83 [95% CI, 0.71;0.92]. It reinforces the reproducible nature of the laparoscopy score and encourages its use according to the recommendations in cases of advanced EOC. Based on our results, the inter-observer concordance of the adjusted Fagotti score, taking into consideration non-explorable areas with extensive carcinomas, was average, with an ICC of 0.64 [95% CI, 0.46; 0.82]. Similarly, the modified Fagotti score had average inter-observer concordance, with an ICC of 0.72 [0.56–0.87]. We identified three parameters that were more often judged by evaluators to be "non-explorable": mesentery involvement, stomach infiltration and liver damage. By removing these parameters, we calculated a so-called explorable Fagotti score. The concordance of this explorable Fagotti score was as good as that of Fagotti's score or even better with an ICC of 0.86 [95% CI, 0.75–0.94]. There had a single patient with Figo 1–2 disease patient. The Fagotti score has been validated for all stages of pathology but we can see this sample is not representative of the different stages. The intra-class coefficient (ICC) was recalculated by excluding this case. Our main conclusion did not differ.

The Italian Olympia-MITO 13 study is the only comparable study we reported^19^. This prospective multicenter study was conducted by the team of Fagotti et al., which was the coordinating center from 2010 to 2012. The objective was to verify the reproducibility of the Fagotti score in the description of intra-abdominal tumor spread. Surgeons working in satellite centers were selected and trained to apply this laparoscopic scoring system. 120 patients who underwent an exploratory laparoscopy in 24 satellite centers were included in per-protocol analysis. 9.5% of videos were rated as being of poor quality in the Olympia-MITO 13 vs. 13.3% in our study. These videos had not been taken into account in their statistical analyses. As in the MITO-13 study, some parameters were rated non-assessable by surgeons.

The management of these non-explorable parameters in scoring had not been addressed by Fagotti's team and there were no other studies found in the literature on this topic. However, it is the exploration of all the anatomical zones described that makes it possible to produce the Fagotti score and estimate predictive resectability. We decided to process the non-explorable data by modulating these parameters and calculating different scores, always with the aim of studying the inter-observer concordance. Working on the assumption that the non-explorable parameters were met we calculated the adjusted Fagotti score. This adjusted Fagotti score had worse inter-observer concordance than Fagotti's score. We were also interested in the modified Fagotti score proposed by the team of Brun et al. ^19^. The inter-observer concordance of this score within our study was average, with a CCI of 0.718 [0.556–0.868]. This score is built on 4 of the 8 parameters that, according to their analysis, best met the criteria of predictive resectability (specificity ≥ 75%, PPV ≥ 50%, and NPV ≥ 50%). These parameters are carcinosis of the diaphragmatic cupolas, retraction of the mesentery, infiltration of the stomach and liver damage. However, in our study it was precisely these parameters (3 out of the 4) that were the least explorable: retraction of the mesentery, infiltration of the stomach and liver damage. This is in line with the data described in the literature. For comparison, in the MITO-13 study carried out by the team of Fagotti et al., evaluation of the mesentery was not possible in 31 cases out of 120 or 25.8% of cases, which is very similar to our findings (19.39%). Stomach infiltration and liver damage were also, along with digestive tract infiltration, the parameters most frequently rated as not explorable. By removing the 3 parameters that were least likely to be able to be assessed from our study, we calculated an explorable Fagotti score. The inter-observer concordance of the explorable Fagotti score was comparable or even better than that of the Fagotti score. Furthermore, we may wonder whether these non-explorable areas might impact decision-making when calculating the Fagotti score for patients with extensive intra-abdominal diffusion of the disease. If we take the threshold of 8, we see that the way in which non-explorable areas are treated results in little difference.

Imaging is less efficient in assessing intra-abdominal resectability than surgery. CT would have a sensitivity of 60–79%, PET 59%, with consistant underestimation of the extent of carcinosis (sensitivity < 30% if < 0.5 cm)^14^. Pinto's study is based on the fact that imaging is not effective in detecting small-volume carcinomatosis. Laparoscopy may directly visualize intraperitoneal involvement, but it has inherent limitations when investigating tumours behind the gastrosplenic ligament, in the lesser sac, mesenteric root or when exploring the retroperitoneum. The major benefit of laparoscopy appears as an ultimate triage step in situations where the imaging diagnosis is uncertain regarding resectability and the presence of diffuse small-volume carcinomatosis^22^.

It is also based on biological tools such as CA-125. The study by Coussy et al. described 68.9% resectability with a median rate of 500 preoperatively and linked the CA-125 level to the percentage of optimal tumor resectability^23^.

The study of Petrillo et al. confirmed that Fagotti’s score was an accurate tool in the prediction of complete PDS in women with EOC^15^. 234 patients underwent an operability laparoscopy accompanied by calculation of the Fagotti score and followed by a cytoreduction laparotomy. The score was used to estimate the chances of a complete cytoreduction surgery (CC0). 137 patients (57.7%) were able to have CC0 surgery. Agreement between laparoscopic and laparotomic findings exceeded 90% for all criteria except for GI (gastrointestinal) infiltration (88.6%). For a score of ≥ 8, the probability of complete surgery upon laparotomy (zero residual tumor) was 8.3% and the rate of unnecessary exploratory laparotomies was 28.3%. For a score ≥ 10, the probability of optimal surgery upon laparotomy (zero residual tumor) was 0 and the rate of unnecessary exploratory laparotomies was 33.2%. The threshold of 10 was proposed for the assessment of complete resectability (NP4).

In this model to predict incomplete cytoreduction in advanced epithelial ovarian cancer by Petrillo, two parameters have been excluded : laparoscopic assessments of mesenteral retraction and militaric carcinomatosis on the serosa of the small bowel are considered as absolute criteria of unresectability. A study by Heitz et al. with 739 patients showed that the residual disease after incomplete cytoreductive surgery was mainly in the mesentery and serosa of the small bowel (79.8%)^15^.

Our study has biases and limitations. First, the surgical technique was not standardized and could differ, depending on the surgeon. The number of videos was low (15) and this may have constituted a bias. Regarding sample size, this is an exploratory pilot study. To calculate the number of subjects required, we need an assumption of the expected correlation coefficient and/or its confidence interval, which we didn't have at this stage. We relied on actual recruitment capacity to carry out the study at a pilot stage. With more subjects, we would have had narrower confidence intervals. In the study for Fagotti and Fagotti Explorable, the confidence intervals have a lower bound of 0.70 and 0.75, which, even in the worst-case scenario, guarantee a good correlation. On the other hand, for the adjusted and modified Fagotti, the lack of power and the need for a larger study are debatable. Laparoscopy video editing also represented an intrinsic bias. The poor or average quality of some laparoscopy recordings could induce measurement bias. In fact, two videos were judged with 45% satisfaction, but they were interpretable by the reviewers. We have chosen to keep them, as they are real-life events which can have an impact on clinical applicability. The strengths of this study include its prospective and multi-centre nature. The study population was representative of patients diagnosed with EOC at surgical centers in France. Selection bias was limited by using initial exploratory laparoscopies, i.e. for initial management only. The number of evaluators tested was large, comprising 11 reviewers in total. All the videos reviewed by each reviewer were identical.

The recommendations suggest that a major retraction of the mesentery, damage to the hail, hepatic pedicle or diffuse miliary involvement, and the need to perform several surgical procedures are a contraindication to a first resectability gesture. Our study was based on the original 2006 Fagotti score. The criteria for non-resectability were redefined retrospectively in line with the ESMO-ESGO consensus 2019. Following these recommendations, Fagotti excluded mesenterial retraction and carcinomatosis on the serosa of the small bowel from the scoring system. The initial step of the laparoscopic assessment process, which focuses on excluding markers of non-resectability^15,19^. Among the intra-abdominal sites explored, the region of the spleen and lesser sac are analyzed if the mesenteric root is not affected. It is interesting to note that in our study, when the mesenteric root was affected, it was associated with a higher number of unexplorable regions (upper abdominal region with liver and stomach).

Finally, it would be interesting to know the surgical outcome of patients in order to compare the evaluation of the Fagotti score and its implication in current practice. However, our study was not based on the score's performance, but on its reproducibility. This could therefore be the subject of a further study.

In conclusion, the present study showed a real external validation of the Fagotti score. The Fagotti score is a tool that has good inter-observer reproducibility. Further studies seem necessary to determine how non-explorable areas of this score should be dealt with.

### Supplementary Information

Supplementary Information.

## Data Availability

All data generated or analysed during this study are included within this published article.
