# Author Correction: First external validity study of the Fagotti score in ovarian cancer

**DOI:** 10.1038/s41598-024-69714-8

**Published:** 2024-08-13

**Authors:** Sarah Aida, Mathieu Levaillant, Henri Azaïs, Marcos Ballester, Geofroy Canlorbe, Pauline Chauvet, Tristan Gauthier, Cyrille Huchon, Yohan Kerbage, Martin Koskas, Lise Lecointre, Lobna Ouldamer, Émilie Raimond, Vincent Lavoué, Guillaume Legendre

**Affiliations:** 1 Department of Gynecology, University Hospital, 49100 Angers, France; 2 Department of Public Health, University Hospital, 49100 Angers, France; 3 Department of Gynecology and Breast Oncological Surgery, Georges‑Pompidou European Hospital, APHP Centre, University of Paris, 75015 Paris, France; 4 Department of Gynecology, University Hospital, Diaconnesses La Croix Simon, 75012 Paris, France; 5 Department of Gynecology, University Hospital, APHP- La Pitié Salpêtrière, 75013 Paris, France; 6 Department of Gynecology, University Hospital, 63000 Clermont‑Ferrand, France; 7 Department of Gynecology, University Hospital, 87042 Limoges, France; 8 Department of Gynecology, Lariboisière Hospital, AP‑HP, University of Paris, 75010 Paris, France; 9 Department of Gynecology, University Hospital, 59000 Lille, France; 10 Department of Gynecology, Hospital, Bichat, Paris Cité University, 75018 Paris, France; 11 Department of Gynecology, University Hospital, 67200 Strasbourg, France; 12 Department of Gynecology, University Hospital, Bretonneau, 37044 Tours, France; 13 Department of Gynecology, University Hospital, 51100 Reims, France; 14 Department of Gynecology, University Hospital, 35033 Rennes, France

Correction to: *Scientific Reports* 10.1038/s41598-024-62568-0, published online 27 May 2024

The original version of this Article contained errors in the names of the authors Sarah Aida, Mathieu Levaillant, Henri Azaïs, Marcos Ballester, Geofroy Canlorbe, Pauline Chauvet, Tristan Gauthier, Cyrille Huchon, Yohan Kerbage, Martin Koskas, Lise Lecointre, Lobna Ouldamer, Émilie Raimond, Vincent Lavoué & Guillaume Legendre, which were incorrectly given as Aida Sarah, Levaillant Mathieu, Azaïs Henri, Ballester Marcos, Canlorbe Geofroy, Chauvet Pauline, Gauthier Tristan, Huchon Cyrille, Kerbage Yohan, Koskas Martin, Lecointre Lise, Ouldamer Lobna, Raimond Émilie, Lavoué Vincent & Legendre Guillaume.

The Author Contribution section now reads:

G.L. provided the initial concept for the trial and was responsible for the methodology supervision, validation, writing the review and editing. M.L. performed the statistical analyses. S.A. contributed to data analysis, writing the original draft and the review. G.C., V.L., H.A., M.K., L.L., P.C., C.H., E.R., T.G., M.B. contributed to patient recruitment. All authors contributed to the drafting of the report and approved the final version for submission.

The original Article has been corrected.

