# Supplementary material for: First external validity study of the Fagotti score in ovarian cancer
Source: Sci Rep. 2024 May 27;14:12133. doi: 10.1038/s41598-024-62568-0 (PMC11130284; doi:10.1038/s41598-024-62568-0)
Supplement: Supplementary file 1 — Supplementary Information. [file 41598_2024_62568_MOESM1_ESM.docx]

Appendix 1. List of hospitals involved in video collection and review

- The University Hospital of Lille

- The University Hospital of Angers

- The Hospital Center of Bichat of the APHP

- The Hospital Center of Lariboisière of the APHP

- The Hospital Center of Pitié of the APHP

- The HEGP Hospital Center

- The University Hospital of Clermont-Ferrand

- The University Hospital of Limoges

- The University Hospital of Reims

- The University Hospital of Rennes

- The University Hospital of Strasbourg

| Variables | Data |
| --- | --- |
| Number of ovarian cancers operated on per year on average, median [interquartile range] | 10 [5-20] |
| Years of experience, *median y [interquartile range]* | 8 [6-11] |
| If a criterion of the Fagotti score is "non-assessable" it is considered to be |  |
| Affected | 27.2 % (3) |
| Not affected | 9.1 % (1) |
| Context-dependent* | 63.6 % (7) |
| *imaging, tumor load |  |

Table S1. Reviewers characteristics
